# Translation and Validation of Modified Dental Anxiety Scale: The Nepali Version

**DOI:** 10.1155/2017/5495643

**Published:** 2017-01-29

**Authors:** Jamal Giri, Prabhat Ranjan Pokharel, Rajesh Gyawali, Bhushan Bhattarai

**Affiliations:** Department of Orthodontics, College of Dental Surgery, B. P. Koirala Institute of Health Sciences, Dharan, Nepal

## Abstract

*Introduction.* For proper management of anxious dental patients it is imperative to assess their levels of dental anxiety before treatment. Modified Dental Anxiety Scale (MDAS) is the most commonly used questionnaire to assess dental anxiety. But a Nepali version of MDAS is still lacking. Hence, the objective of this study was to develop a reliable and valid Nepali version of MDAS.* Materials and Methods.* The English version of the MDAS was translated into Nepali following a forward and backward translation process. Following pretesting and cognitive interviewing a final version of Nepali questionnaire was obtained. One hundred and fifty patients attending Department of Orthodontics completed the Nepali version of MDAS questionnaire at their convenience. Also, patients were asked to rate their overall anxiety on a 100 mm visual analog scale (VAS). A test-retest of the questionnaire was performed with 30 patients after 2 weeks.* Results.* Cronbach's alpha value of the Nepali version of MDAS was 0.775. The Intraclass Correlation Coefficient between test and retest was 0.872. Spearman's correlation coefficient between the total MDAS score and VAS score was 0.838.* Conclusion.* The translated Nepali version of MDAS is a reliable and valid instrument to measure the dental anxiety of Nepali patients.

## 1. Introduction

Anxiety is a natural human emotion encountered in various situations; dental treatment is no exception. Dental anxiety is defined as patient's response to stress that is specific to the dental situation [1]. It is a common reason for people avoiding dental treatment [2]. Dental patients with high level of anxiety present a major management problem [3]. Hence, for proper management of anxious patients it is imperative to assess their levels of dental anxiety before treatment.

Several questionnaire based scales have been developed to assess the dental anxiety of patients. Modified Dental Anxiety Scale (MDAS) [4], Corah's Dental Anxiety Scale [5], Dental Fear Survey [6], General Geer Fear Scale [7], and so forth are some commonly used scales for assessing the level of anxiety of dental patients. MDAS is the most commonly used questionnaire which is a modification of Corah's Dental Anxiety Scale.

MDAS comprises 5 questions, each assessing the dental anxiety levels in different dental situations. All questions have 5 responses in Likert scale ranging from “not anxious” to “extremely anxious.” Each response is scored from 1 to 5. So, a “not anxious” response is scored 1 and an “extremely anxious” response is scored 5. For assessing the level of dental anxiety of the patient, response scores of all 5 questions are added. The total score of this scale ranges from 5 to 25 with cut-off scores 14 and 19 suggestive of high dental anxiety and dental phobia, respectively [8]. MDAS questionnaire is relatively simple to complete and less time consuming. More importantly, the process of completion does not raise the patient's anxiety level [9]. MDAS has been translated into different languages, namely, Arabic, Chinese, Greek, Romanian, Spanish, Tamil, Turkish, and Italian [10]. It has a good cross-cultural reliability and validity [11]. But a Nepali version of MDAS is still lacking. The aim of this study was to develop a reliable and valid Nepali version of MDAS so that it can be used as an assessment tool to assess dental anxiety of Nepali patients.

## 2. Materials and Methods

This cross-sectional study was conducted among dental patients who reported to Department of Orthodontics, B. P. Koirala Institute of Health Sciences (BPKIHS), Dharan, Nepal, from December 2015 to January 2016. The study was approved by ethical review board of BPKIHS.

### 2.1. Instrument

The English version of the MDAS was translated following forward and backward translation process. The English version of the questionnaire was translated into Nepali by two independent bilingual professionals. The Nepali versions thus obtained were back translated into English by two other translators who were blinded to the original English version of the questionnaire. The back translated versions were reviewed and with the help of the bilingual professionals, a reconciled Nepali version of the questionnaire was obtained. The Nepali version of the questionnaire was pretested among 30 dental patients. The patients were systematically debriefed and interviewed in a waiting room of the department by the principal investigator (JG) while they were completing the questionnaire. Following the pretesting and cognitive interviewing a final version of the questionnaire in the target language (Nepali) was obtained. The final version was referred to as MDAS-N.

### 2.2. Participants

One hundred and fifty patients attending Department of Orthodontics of BPKIHS were enrolled in this study following a nonprobability convenient sampling method. Patients who gave history of dental treatment were excluded from the study. Informed consent was obtained from all the patients. Demographic data of the patients were recorded. Then, the patients were requested to complete the MDAS-N questionnaire in the waiting room of the department at their convenience. On completion of the questionnaire patients were asked to rate their overall anxiety on 100 mm visual analog scale (VAS). Further, 30 patients recompleted the questionnaire after 2 weeks of initial completion.

### 2.3. Statistical Analysis

Data were analyzed using SPSS version 11 statistical package. Pertinent descriptive statistics were calculated. Internal consistency reliability of MDAS-N was measured using Cronbach's alpha. Sampling adequacy was tested using Kaiser–Meyer–Olkin (KMO) test. Bartlett's test of sphericity was employed to determine the suitability of the data for factor analysis. Principal component factor analysis was performed. Intraclass Correlation Coefficient (ICC) was used to assess the test-retest agreement of completed questionnaires. Spearman's correlation coefficient between MDAS-N score and VAS score was calculated to assess the convergent validity. Independent samples *t*-test was used to compare the mean MDAS score values of male and female samples.

## 3. Results

### 3.1. Descriptive Statistics

Out of the 150 patients who completed the questionnaire, 60% were females. The average age of the patients was 20.93 ± 6.01 years (range: 16–42 years). The response rate was 100% with all the questionnaire forms duly filled. Thirty retest questionnaires also displayed 100% response rate.  Table 1 depicts the categorization of the patients based on their total MDAS. Two percent of the patients were found to be extremely anxious with MDAS ≥ 19 and 20.67% demonstrated high dental anxiety.

### 3.2. Reliability Measures

Cronbach's alpha of the Nepali version of MDAS was 0.775 which shows good internal consistency of the instrument.  Table 2 depicts the interitem correlation matrix and Table 3 shows Cronbach's alpha if item is deleted for 5 items and corrected item-total correlation. Deletion of any item did not increase Cronbach's alpha value. The corrected item-total correlation ranged between 0.445 and 0.64.

The test-retest reliability was calculated from the scale scores of 30 patients who recompleted the questionnaire 2 weeks after the initial completion. Intraclass Correlation Coefficient (ICC) values were calculated for five individual items and total score. The test-retest ICC of the total score was 0.872 (*P* < 0.001) suggestive of almost perfect agreement (Table 4).

### 3.3. Validity Measures

**Figure 1:**
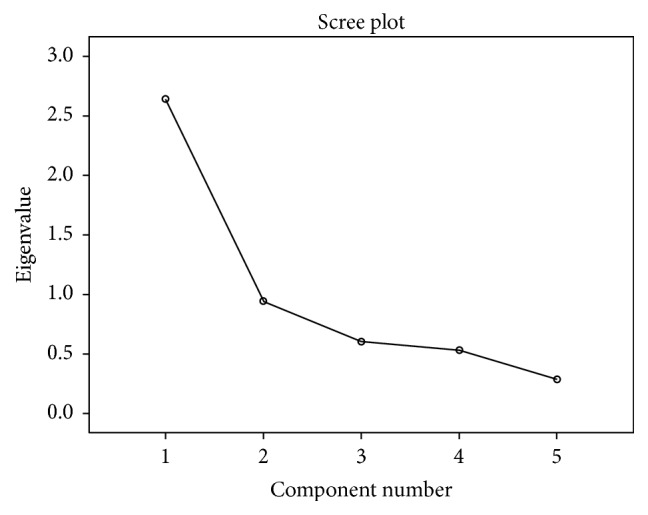
Scree plot displaying a single component with eigenvalue > 1.

The value of KMO measure of sampling adequacy was 0.729 which is satisfactory and Bartlett's test of sphericity was statistically significant (*χ*^2^ = 218.05, *P* < 0.001). Hence, the data was deemed suitable for factor analysis. Results of the principal component factor analysis revealed a single significant component accounting for 52.841% of total variance with an eigenvalue of 2.642 (Figure 1). All component loadings were > 0.7 except for item 4 (Table 5).

Convergent validity of the Nepali version of MDAS was assessed using Spearman's correlation coefficient. Spearman's correlation coefficient between the total MDAS score and VAS score was 0.838 (*P* < 0.01), indicative of strong positive correlation. But there was no statistically significant difference between the mean MDAS scores of male and female samples (*t* = 0.450, df = 148, and *P* = 0.653).

## 4. Discussion

An appropriate instrument to assess the dental anxiety of Nepali population was lacking. Hence, this hospital based cross-sectional study was conducted to develop an appropriate Nepali version of MDAS and evaluate the psychometric properties (reliability and validity) of the instrument. This newly developed Nepali version of MDAS is suitable for use among dental patients due to its good internal consistency reliability (Cronbach's alpha = 0.775), high test-retest reliability (Intraclass Correlation Coefficient = 0.872), and excellent convergent validity (Spearman's correlation coefficient = 0.851). The high completion rate (100%) of this questionnaire also suggests that it is easy to complete with minimal supervision.

The mean MDAS score of the Nepali samples of this study was slightly higher than that of the community samples of the original study conducted in United Kingdom (12.28 ± 3.02 versus 10.36 ± 5.36) [4]. But it was similar to the mean MDAS score of adult female samples of the original study (12.87 ± 5.6) [4]. Nevertheless, these mean MDAS scores are suggestive of moderate level of dental anxiety prevalent among both of the study samples (Nepal and United Kingdom). Extreme dental anxiety (MDAS score ≥ 19) was prevalent among 2% of the samples of this study. Similar levels of dental anxiety have been reported in studies conducted in India [1] and Finland [11]. However, there are studies which have reported higher prevalence of extreme dental anxiety or dental phobia [12–14]. This low prevalence of extreme dental anxiety among Nepali samples could be attributed to ethnic differences. Another possible explanation for this is that the Nepali samples were patients seeking dental treatment. The level of extreme dental anxiety could be more among general Nepali population. This is an important issue for future research.

Cronbach's alpha coefficient for Nepali version of MDAS was found to be 0.775 which is similar to Cronbach's alpha value of Indian version (0.78) [15]. However, this value is less than that of community samples of the original English version (0.9) [4]. Cronbach's alpha value obtained for Arabic (0.87) [13], Italian (0.92) [14], Spanish (0.88) [16], and Greek (0.9) [17] versions was also greater compared to this value. But it is still above accepted Cronbach's alpha value of 0.7 and is suggestive of good internal consistency reliability [18]. The corrected item-total correlation coefficients of all 5 items were above 0.4. This shows that the items fitted well with each other in the instrument. There was no increase in Cronbach's alpha value on deletion of any items which vindicated the inclusion of the items in the instrument. The instrument also demonstrated excellent test-retest reliability (ICC = 0.872).

Validity is another integral psychometric property of any instrument. Principal component analysis was used to assess the construct validity. Only one component emerged from the principal component analysis and factor loading for all 5 items of the instrument was above 0.3. It is evident that all 5 items are contributing meaningfully to the emerged component. Furthermore, there was a strong positive correlation between MDAS score and VAS score. Patients who had high MDAS score also had high VAS score. Such a high correlation between this instrument and VAS, which is an established instrument [19], is suggestive of excellent convergent validity of this instrument.

Many studies have found that females are more anxious about dental treatment compared to their male counterparts [20, 21]. It is believed that the physiological emotions such as social phobia, panic, depression, stress, and fear are more common in females and females are more willing to admit to symptoms of anxiety [22]. However, the results of this study indicate that the level of dental anxiety is independent of gender of Nepali patients. The mean MDAS scores of male and female patients were 12.15 ± 2.99 and 12.38 ± 3.06, respectively, and the difference was not found to be statistically significant. This could be attributed to ethnic and cultural differences of Nepali females. This is an interesting finding and needs to be verified with further studies with larger sample size.

Association between chronological age and level of dental anxiety of patients is still unclear. Studies by Humphris et al. [12] and Acharya [15] have found that younger patients were more anxious than older patients. But Tunc et al. [23] have found higher levels of dental anxiety among older patients. However, there are studies which have found no association between chronological age and level of dental anxiety [21, 24, 25]. This study could not elucidate the relationship between chronological age and the level of dental anxiety owing to a narrow range of chronological age of the samples (16 to 42 years). Further studies with wider range of chronological age of Nepali samples and preferably longitudinal studies will help us understand this relationship.

There are few limitations of this study. A written questionnaire was used to assess the dental anxiety of the patients. Hence, the level of dental anxiety of illiterate patients could not be assessed. Also, the study was hospital based; it does not represent the level of dental anxiety of general Nepali population. Hence, the findings of this study cannot be extrapolated to the entire Nepali population.

Further studies on level of dental anxiety among Nepali population may be conducted using this Nepali version of MDAS. Understanding of level of dental anxiety among the population will help the clinicians develop treatment protocols which will help to alleviate the dental anxiety of patients. This study further supports the statement that the MDAS scale displays good cross-cultural reliability and validity.

## 5. Conclusion

The translated Nepali version of Modified Dental Anxiety Scale (MDAS-N) is a reliable and valid instrument to measure the dental anxiety of Nepali patients.

## Figures and Tables

**Table 1:** Categorization of patients based on MDAS scores.

MDAS score range	Percentage/number	Mean MDAS score
0–5 (not anxious)	0% (0)	—
6–10 (low anxiety)	26% (39)	8.72 ± 1.28
11–14 (moderate anxiety)	51.33% (77)	12.27 ± 1.03
15–18 (high anxiety)	20.67% (31)	15.90 ± 1.11
19–25 (extreme anxiety/phobic)	2% (3)	21.67 ± 3.06
Total patients	100% (150)	12.29 ± 3.03

MDAS: Modified Dental Anxiety Scale.

**Table 2:** Interitem correlation matrix.

Item number	Q1	Q2	Q3	Q4	Q5
Q1	1.000	.465	.397	.386	.409
Q2	.465	1.000	.385	.429	.321
Q3	.397	.385	1.000	.306	.709
Q4	.386	.429	.306	1.000	.273
Q5	.409	.321	.709	.273	1.000

**Table 3:** Item-total statistics.

Item number	Scale mean if item deleted	Scale variance if item deleted	Corrected item-total correlation	Squared multiple correlation	Cronbach's alpha if item deleted
Q1	10.3867	6.467	.550	.322	.734
Q2	10.4200	6.420	.518	.319	.744
Q3	8.8733	5.373	.640	.536	.701
Q4	10.8867	7.135	.445	.239	.766
Q5	8.5800	5.561	.607	.522	.713

**Table 4:** Intraclass Correlation Coefficient (ICC) values for test-retest reliability of the five items and total score of Nepali version of MDAS.

MDAS item number	ICC	95% CI		*F*-test value	*P* value
		Lower	Upper		
(1)	0.721	0.375	0.873	8.153	<0.001
(2)	0.695	0.329	0.860	7.394	<0.001
(3)	0.848	0.658	0.930	14.678	<0.001
(4)	0.682	0.434	0.835	5.25	<0.001
(5)	0.873	0.749	0.937	14.244	<0.001

Total score	0.872	0.530	0.953	23.209	<0.001

MDAS: Modified Dental Anxiety Scale.

**Table 5:** Component matrix.

Items	Matrix of the factorial structure	Communalities
Q1	0.730	0.533
Q2	0.707	0.499
Q3	0.790	0.625
Q4	0.631	0.398
Q5	0.766	0.587

## Abbreviations

MDAS: Modified Dental Anxiety Scale
MDAS-N: Modified Dental Anxiety Scale (Nepali version)
VAS: Visual analog scale.

## References

[1] Appukuttan D., Datchnamurthy M., Deborah S. P., Hirudayaraj G. J., Tadepalli A., Victor D. J. (2012). Reliability and validity of the Tamil version of modified dental anxiety scale. *Journal of Oral Science*.

[2] Ilgüy D., Ilgüy M., Dinçer S., Bayirli G. (2005). Reliability and validity of the modified dental anxiety scale in Turkish patients. *Journal of International Medical Research*.

[3] Appukuttan D. P. (2016). Strategies to manage patients with dental anxiety and dental phobia: literature review. *Clinical, Cosmetic and Investigational Dentistry*.

[4] Humphris G. M., Morrison T., Lindsay S. J. (1995). The modified dental anxiety scale: validation and United Kingdom norms. *Community Dental Health*.

[5] Corah N. L. (1969). Development of a dental anxiety scale. *Journal of Dental Research*.

[6] Kleinknecht R. A., Thorndike R. M., McGlynn F. D., Harkavy J. (1984). Factor analysis of the dental fear survey with cross-validation. *The Journal of the American Dental Association*.

[7] Geer J. H. (1965). The development of a scale to measure fear. *Behaviour Research and Therapy*.

[8] Humphris G., Crawford J. R., Hill K., Gilbert A., Freeman R. (2013). UK population norms for the modified dental anxiety scale with percentile calculator: adult dental health survey 2009 results. *BMC Oral Health*.

[9] Humphris G. M., Hull P. (2007). Do dental anxiety questionnaires raise anxiety in dentally anxious adult patients? A Two-Wave Panel Study. *Primary Dental Care: Journal of the Faculty of General Dental Practitioners (UK)*.

[10] Sitheeque M., Massoud M., Yahya S., Humphris G. (2015). Validation of the Malay version of the Modified Dental Anxiety Scale and the prevalence of dental anxiety in a Malaysian population. *Journal of Investigative and Clinical Dentistry*.

[11] Humphris G. M., Freeman R., Campbell J., Tuutti H., D'Souza V. (2000). Further evidence for the reliability and validity of the Modified Dental Anxiety Scale. *International Dental Journal*.

[12] Humphris G. M., Dyer T. A., Robinson P. G. (2009). The modified dental anxiety scale: UK general public population norms in 2008 with further psychometrics and effects of age. *BMC Oral Health*.

[13] Abu-Ghazaleh S. B., Rajab L. D., Sonbol H. N., Aljafari A. K., Elkarmi R. F., Humphris G. (2011). The Arabic version of the modified dental anxiety scale. *Saudi Medical Journal*.

[14] Facco E., Gumirato E., Humphris G. (2015). Modified dental anxiety scale: validation of the Italian version. *Minerva Stomatologica*.

[15] Acharya S. (2008). Factors affecting dental anxiety and beliefs in an Indian population. *Journal of Oral Rehabilitation*.

[16] Coolidge T., Hillstead M. B., Farjo N., Weinstein P., Coldwell S. E. (2010). Additional psychometric data for the spanish modified dental anxiety scale, and psychometric data for a spanish version of the revised dental beliefs survey. *BMC Oral Health*.

[17] Coolidge T., Arapostathis K. N., Emmanouil D. (2008). Psychometric properties of Greek versions of the Modified Corah Dental Anxiety Scale (MDAS) and the Dental Fear Survey (DFS). *BMC Oral Health*.

[18] Tavakol M., Dennick R. (2011). Making sense of Cronbach's alpha. *International Journal of Medical Education*.

[19] Facco E., Zanette G., Favero L. (2011). Toward the validation of visual analogue scale for anxiety. *Anesthesia Progress*.

[20] Gupta G., Shanbhag N., Puranik M. P. (2015). Cross-cultural adaptation of Kannada version of modified dental anxiety scale among an adult Indian population. *Journal of Clinical and Diagnostic Research*.

[21] Saatchi M., Abtahi M., Mohammadi G., Mirdamadi M., Binandeh E. (2015). The prevalence of dental anxiety and fear in patients referred to Isfahan Dental School, Iran. *Dental Research Journal*.

[22] Reynolds C. R. (1998). Need we measure anxiety differently for males and females?. *Journal of Personality Assessment*.

[23] Tunc E. P., Firat D., Onur O. D., Sar V. (2005). Reliability and validity of the Modified Dental Anxiety Scale (MDAS) in a Turkish population. *Community Dentistry and Oral Epidemiology*.

[24] Gisler V., Bassetti R., Mericske-Stern R., Bayer S., Enkling N. (2012). A cross-sectional analysis of the prevalence of dental anxiety and its relation to the oral health-related quality of life in patients with dental treatment needs at a university clinic in Switzerland. *Gerodontology*.

[25] Malvania E. A., Ajithkrishnan C. G. (2011). Prevalence and socio-demographic correlates of dental anxiety among a group of adult patients attending a dental institution in Vadodara city, Gujarat, India. *Indian Journal of Dental Research*.

